# The development of trunk control and its relation to reaching in infancy: a longitudinal study

**DOI:** 10.3389/fnhum.2015.00094

**Published:** 2015-02-24

**Authors:** Jaya Rachwani, Victor Santamaria, Sandra L. Saavedra, Marjorie H. Woollacott

**Affiliations:** ^1^Human Physiology and Institute of Neuroscience, University of OregonEugene, OR, USA; ^2^Department of Rehabilitation Sciences, University of HartfordWest Hartford, CT, USA

**Keywords:** infant development, independent sitting, posture, trunk control, reaching, EMG

## Abstract

The development of reaching is crucially dependent on the progressive control of the trunk, yet their interrelation has not been addressed in detail. Previous studies on seated reaching evaluated infants during fully supported or unsupported conditions; however, trunk control is progressively developed, starting from the cervical/thoracic followed by the lumbar/pelvic regions for the acquisition of independent sitting. Providing external trunk support at different levels to test the effects of controlling the upper and lower regions of the trunk on reaching provides insight into the mechanisms by which trunk control impacts reaching in infants. Ten healthy infants were recruited at 2.5 months of age and tested longitudinally, until 8 months. During the reaching test, infants were placed in an upright seated position and an adjustable support device provided trunk fixation at pelvic and thoracic levels. Kinematic and electromyographic data were collected. Results showed that prior to independent sitting, postural instability was higher when infants were provided with pelvic compared to thoracic support. Associated reaches were more circuitous, less smooth and less efficient. In response to the instability, there was increased postural muscle activity and arm muscle co-activation. Differences between levels of support were not observed once infants acquired independent sitting. These results suggest that trunk control is acquired in a segmental sequence across the development of upright sitting, and it is tightly correlated with reaching performance.

## Introduction

Sitting postural control and reaching are distinguishable yet inter-related motor milestones, which are progressively acquired during the first years of life. When tasks require reaching while sitting, a simple reach toward an object involves complex interaction of musculoskeletal and neural systems to optimize the movement. Moreover, the emergence of posture and reaching skills is critical to subsequent perceptual, cognitive and social development (Sommerville et al., 2005; Soska et al., 2010; Lobo and Galloway, 2012).

The relation between posture and arm movements is evident in neonates. When newborns are fully supported, either in a reclined or upright sitting position, their usually chaotic arm movements are more coordinated and directed, indicating that pre-reaching movements are influenced by posture (Von Hofsten, 1982; Amiel-Tison and Grenier, 1983). However, newborns may not actually want to access the toy but rather pre-reaching movements may function to orient the infants' attention to the goal (Von Hofsten, 1982; Campos et al., 2008). In addition, the hands also attract considerable amount of attention and newborns will work to keep their hands in view (Van der Meer, 1997).

Beginning at 3 months, arm extensions are gradually replaced by goal directed reaches that are mainly unsuccessful in grasping the object. Grasping is typically achieved at the age of 4 months (Van der Fits et al., 1999a; De Graaf-Peters et al., 2007); however, arm movements are jerky with non-linear trajectories and have many movement units (defined as the number of accelerations and decelerations within the velocity profile of the reach (Von Hofsten, 1991). From this age there is an improvement in reaching kinematics and 6 month-old infants develop a straight arm trajectory accompanied by fewer movement units (Von Hofsten, 1991). During this phase of reaching development, there are many factors that influence arm trajectory, including visual perception, neuromuscular forces, biomechanical factors and proprioceptive information. However, the development of postural control for maintaining stability during reaching is indispensable (Bertenthal and Von Hofsten, 1998).

In this regard, 4 month old infants show a functional preference for stabilizing the head while reaching by first activating the neck muscles followed by trunk muscles. Control of the head enables infants to maintain stable vision of the target while reaching (Thelen and Spencer, 1998). Adults use a combination of strategies to attenuate head movement during dynamic tasks (Assaiante and Amblard, 1995; Keshner et al., 1999). However, infants must learn to coordinate head stability with arm movements. At 2 months of age head movements and arm movements are highly coupled (Von Hofsten, 1984; Von Hofsten and Rönnqvist, 1993). From 2 through 4 months of age there is an increased uncoupling of head and arm, allowing more flexibility of eye, head and hand coordination. This uncoupling of head and arm is important for environmental exploration (Hadders-Algra, 2008) and is a precursor of successful reaching (De Graaf-Peters et al., 2007; Van Balen et al., 2012).

Reaching and exploratory behaviors also depend on biomechanical and gravitational forces. Lying supine or prone limits manual exploration whereas sitting creates an advantageous setting for exploring objects (Out et al., 1998; Soska and Adolph, 2014). Within a sitting posture, the inability to sit independently reduces the amount of time the infant invests in exploring the toy because infants need their hands for stability (Harbourne et al., 2013). Nevertheless, when non-sitters are provided with pelvic support, reaching coordination and arm kinematics are significantly improved (Rochat and Goubet, 1995; Hopkins and Rönnqvist, 2002).

In summary, because reaching requires “whole body engagement” (Rochat and Goubet, 1995), its behavior is highly dependent on posture. At about 3 months, when arm extensions are being replaced by goal directed reaches but upright sitting is not mastered, infant reaching is better with external support. As infants generate the ability to sit independently, reaching becomes more coordinated. These observations are also evident in children with cerebral palsy who have deficits in postural control (Van der Heide et al., 2005). Although the progression of postural control is integral to development of reaching, the nature of this interrelation is unknown.

Postural control develops following a cranial-caudal progression, starting with head stabilization on the trunk, occurring at about 2 or 3 months of age. This provides a stable frame of reference for reaching (Assaiante, 1998; Thelen and Spencer, 1998). Control of shoulder and thoracic musculature around 4–5 months enables infants to maintain stability and counteract the reactive forces generated by the forward extension of the arm to successfully reach (Hopkins and Rönnqvist, 2002). As infants gain increasing control of the head and upper trunk, they progress from prop sitting to sitting without support (Harbourne et al., 2013). Subsequently, the control of the lower trunk, pelvis and leg muscles allows them to maintain the center of mass within a stable base of support (Von Hofsten and Woollacott, 1989; Assaiante, 1998; Van der Fits et al., 1999a; Harbourne et al., 2013). Thus, there is a cephalo-caudal development of control of an increasing number of trunk segments for sitting (Butler et al., 2010; Saavedra et al., 2012; Rachwani et al., 2013). However, previous studies have used supine (Van der Fits et al., 1999a; De Graaf-Peters et al., 2007), fully supported (Thelen et al., 1996; Thelen and Spencer, 1998; Van Balen et al., 2012) or unsupported (Van der Fits et al., 1999b; Harbourne et al., 2013) sitting conditions and thus have not examined the effect of trunk support on reaching in the upright position. Here we apply a systematic approach to examine the influence of segmental progression of trunk control on reaching.

In a previous cross-sectional study, we tested upright sitting conditions with trunk support at thoracic and pelvic levels to address contributions of higher and lower regions of the trunk to reaching (Rachwani et al., 2013). We showed that in non-sitters, postural and reaching kinematics depended on the external level of trunk support provided. With thoracic support, postural stability and reaching in non-sitters and sitters did not differ. On the contrary, with pelvic support, sitters outperformed non-sitters. Thus, reaching movement coordination depends on the extent of sitting control. However, the cross-sectional design of the study limited explanation of the causal effects of sitting posture on reaching. Cross sectional studies do not inform us about the mechanisms and trajectory of change and thus results cannot be translated into rehabilitation efforts.

To address this knowledge gap we applied the same experimental paradigm in a longitudinal design, examining intra-individual behavioral and kinematic changes of posture and reaching in conjunction with electromyography (EMG) recordings. As in our previous study we provided two levels of external support during an upright seated reaching task. With the use of video-coding software, we differentiated goal-directed, successful reaches from early pre-reaching movements. Quality of motor performance was assessed with kinematic variables including postural sway and reaching characteristics. Better reaches are more direct (i.e., a straighter reach), smoother (i.e., fewer movement units), more efficient (i.e., lower jerk score), and less reliant on on-line feedback for movement correction (i.e., the peak velocity of the reach occurs closer to the end of the reach) (Wu et al., 2000; Berthier and Keen, 2006).

Postural neuromuscular patterns for reaching include both anticipatory and compensatory adjustments. The role of anticipatory adjustments is to produce a preparatory muscular contraction to stabilize the body in advance (Aruin and Latash, 1995). Compensatory adjustments restore stability after a postural perturbation has occurred (Macpherson et al., 1989). To understand the mechanisms of change for infants, we recorded EMG from postural and arm muscles and documented anticipatory and compensatory activations.

We hypothesized that prior to independent sitting: (1) infants would demonstrate postural instability while reaching with pelvic support compared to thoracic support; (2) postural instability would be accompanied by inefficient and inaccurate reaching; and (3) frequency of anticipatory and compensatory postural reactions would increase as infants acquired independent sitting (Van der Fits et al., 1999a,b) but during pre-sitting stages, activation would be greater with more challenging postural conditions. As infants acquired independent sitting, we expected them to demonstrate invariable reaching and neuromuscular patterns irrespective of the level of support.

## Material and methods

### Participants

Eleven infants were recruited for this study, 1 dropped out after the first session, and 10 infants completed the full protocol. All infants were born at term (5 males and 5 females) and had no known sensory or motor problems. All parents were fluent in English and most of the parents attended ongoing community based parenting groups. Infants began the study at a mean age of 2.5 months (± SD: 0.5 months) and were tested twice a month until the age of 8 months. Infants participated in 10–12 sessions depending on age at entry to the study. If an infant missed an appointment or was fussy they were asked to make up that appointment the following week. Thus, most appointments were 2 weeks apart; however some were 1 or 3 weeks apart. The recruitment was carried out by using flyers in different child care centers in Eugene and Springfield (Oregon, USA). All procedures were approved by the Institutional Review Board for Human Subjects Research at the University of Oregon.

### Materials and procedures

Subjects were asked to come to the laboratory for 120 min sessions. At the first visit, parents were asked to respond to a health questionnaire about their infant, they were informed about the experimental procedure and were asked to sign the informed consent. During each visit, in addition to the reaching test, infants were clinically tested with the Segmental Assessment of Trunk Control (SATCo; Butler et al., 2010) to determine the level of intrinsic trunk control acquired, the Alberta Infant Motor Scale (AIMS; Piper and Darrah, 1994) and the motor subscales of the Bayley Scales of Infant and Toddler Development, 3rd edition (Bayley, 2005) to verify the typical trajectory of gross and fine motor functions. All infants were video recorded during each assessment. The AIMS test was used to determine the onset of independent sitting. The first two data sets when the infant was able to sit without arm support (item 8 from the sitting subscale of the AIMS test) served as the reference point for the developmental timeline. In this item, a specific duration of sitting without arm support was not needed but instead infants were required to demonstrate the ability to be left alone in the sitting position and to move their arms freely or play with a toy. Each infant's data were adjusted to this reference point (time in months of sitting onset = 0). Comparisons were made during the months prior to and after the month of sitting onset. In addition, parents were asked to do the Timed Sitting test twice per week at home to corroborate the onset of independent sitting ability. In this test parents placed the child in sitting with legs in front and timed how long they could stay upright with hands free. Table 1 shows the clinical scores of all subjects (collapsed across 2 sessions at each month with respect to sitting onset).

**Table 1:** **Clinical scores across development**.

	**−4 Months**	**−3 Months**	**−2 Months**	**−1 Month**	**Sitting onset**	**1 Month**	**2 Months**
SATCo score	1.43	2.44	3.77	4.81	6.55	7.83	8.00
(*min–max*)	(1–2)	(1–4)	(2–6)	(4–8)	(4–8)	(6–8)	(8–8)
AIMS	6.71	9.89	16.36	25.52	31.10	37.72	44.33
(*min–max*)	(3–10)	(4–20)	(7–23)	(14–33)	(22–47)	(26–50)	(35–51)
Bayleys: gross motor	5.57	12.78	20.67	26.00	29.00	31.39	33.33
(*min–max*)	(1–11)	(4–23)	(11–27)	(18–33)	(21–36)	(24–36)	(26–37)
Bayleys: fine motor	6.43	10.00	15.05	18.52	20.80	23.61	25.44
(*min–max*)	(4–9)	(7–14)	(7–24)	(11–25)	(16–25)	(19–27)	(23–28)
Age (months)	2.69	3.29	4.11	5.00	5.95	6.76	7.55
(*min–max*)	(2–4)	(2–5)	(3–6)	(4–7)	(4–8)	(5–8)	(6-8)

#### Segmental Assessment of Trunk Control (SATCo)

The SATCo is a clinical measure that examines balance control of the trunk while the evaluator manually supports the trunk at various levels, following a top-down sequence. The evaluator starts by supporting the trunk at a high level, at the shoulder girdle to assess cervical (head) control, through support at the axillae (upper thoracic control), inferior scapula (mid-thoracic control), lower ribs (lower thoracic control), below ribs (upper lumbar control), pelvis (lower lumbar control), and finally, no support, in order to measure full trunk control. During each level of manual support, the test is designed to assess: (1) static control (maintaining a neutral trunk posture) (2) active or anticipatory control (maintaining a neutral posture during head turning or reaching) and (3) reactive control (maintaining or regaining trunk control following a threat to balance, produced by a brisk nudge). The infant's ability to maintain or quickly regain a vertical position of the free region of the trunk in all planes during the assessment of static, active and reactive testing is scored as present or absent. The score reflects the region where infants lose control of posture: a score of 1 = loss of control at the head level, 2 = upper thoracic, 3 = mid-thoracic, 4 = lower thoracic, 5 = upper lumbar, 6 = lower lumbar, 7 = pelvis, 8 = no loss of trunk control (Butler et al., 2010). Thus, the SATCo follows a Guttman scaling, meaning that if an infant has a SATCo score of 4, he/she loses control of posture in static, active or reactive tests when the evaluator supports the lower thoracic region of the trunk but does not lose control of posture when being supported at the levels above that region. This test has been shown to be a valid and reliable measure of the development of trunk control in infants (Butler et al., 2010).

#### Reaching test

The reaching test was conducted with support at thoracic and pelvic levels for every session. The support at the thoracic level was placed below the scapular girdle, and the pelvic level of support was surrounding the pelvis, corresponding to the middle thoracic level and lower lumbar level of the SATCo, respectively. The design of the study was counterbalanced for the first session and was evaluated using the same order throughout the longitudinal process for each infant, with half the infants first being provided with thoracic support, and half first being provided with pelvic support, to eliminate fatigue or training effects as confounding variables.

**Figure 1:**
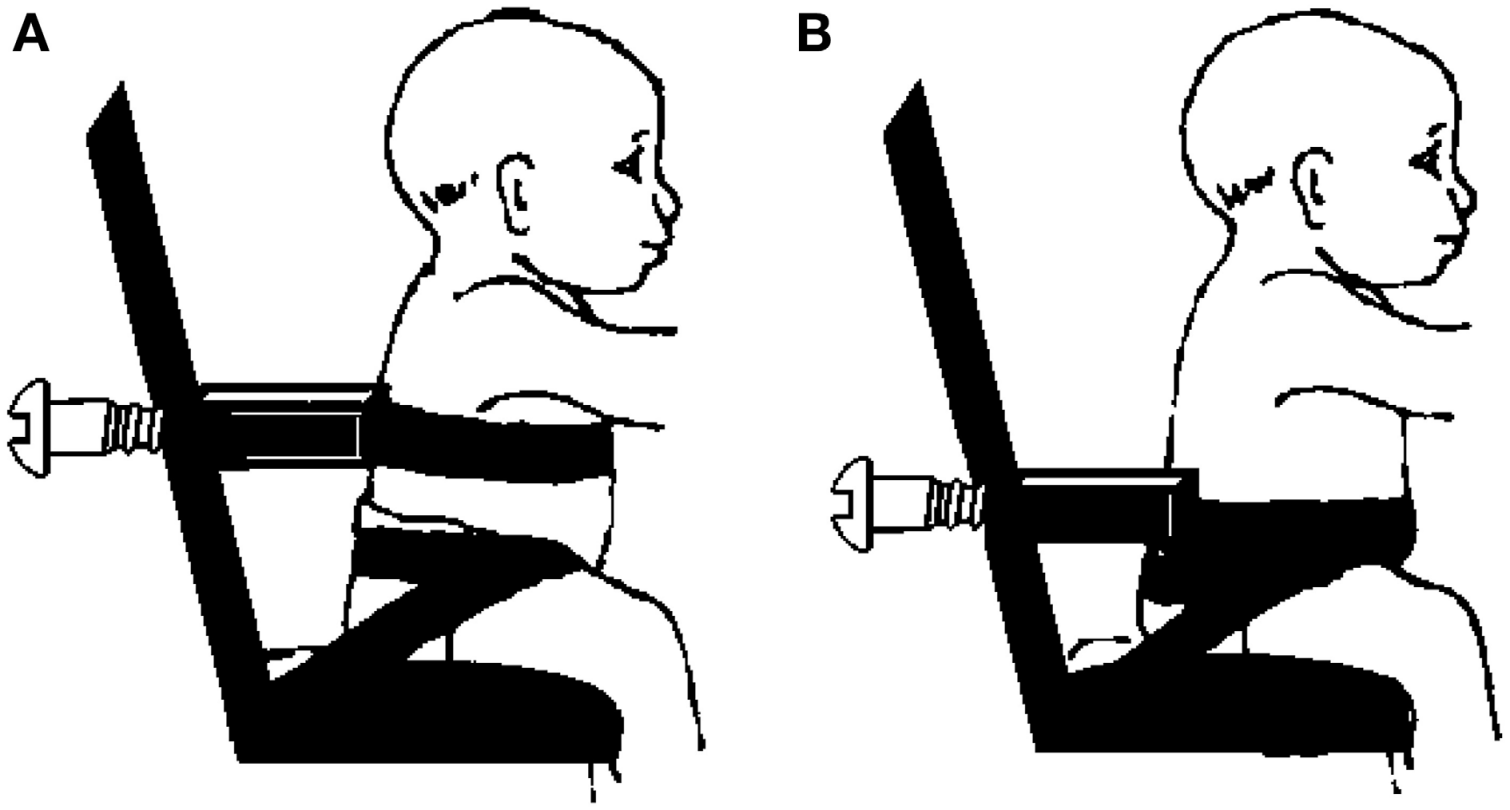
**Schematic representation of infant chair attached to external support device at (A) thoracic and (B) pelvic levels of trunk support**.

The reaching test involved the infant being placed in a seated position on a customized infant chair. The hips of the infant were secured to the chair with specially designed straps and Velcro: two straps were used to wrap each hip joint and the third surrounded both posterior superior iliac spines (Butler et al., 2010). A rigid U-shaped posterior support attached to the back of the chair circled the trunk and provided upright stability of the trunk below the level of interest. The reclined position of the infant chair was used as a safety device in the backwards direction, for securing the infants if they fell backwards. The posterior support was adjusted to allow evaluation of different trunk segments: thoracic and pelvic (Figure 1).

Once posture was stabilized, a colorful toy (colored ring) was presented at approximately the infant's arm length in front of their sternum. The toy was presented using a device placed over the infant's chair that consists of a horizontal brace made of fiberglass with an attachment for the toy. This attachment permits the measurement of the distance from the toy to the chest (anteroposterior axis) and calibration of the height of the toy at the sternum level (vertical axis). Once the exact distance was measured, a toy attached to a rod was placed in the device and was introduced and removed by the tester from the top to the infant's visual field for every trial. The toy was presented approximately 10 times per level of support, but there were occasions in which this number had to be reduced due to fussiness of the infant. If that was the case, the infant's maximum number of trials was noted and the rest of the trials were counted as missing data.

The reaching test was synchronized with the collection of kinematic data (sampling rate = 84 Hz) using magnetic tracking (Minibird system, Ascension Technology, Burlington, VT) and with a 16-channel electromyography (EMG) system (MA300, Motion Lab Systems, Baton Rouge, LA), (sampling rate = 1000 Hz) and video data (sampling rate = 60 Hz).

#### Kinematics

To document the quality of motor performance, four magnetic tracking sensors were placed on the infant: one superficial to the styloid process of the radius on each wrist, one on the posterior and prominent part of the cervical vertebra 7 (c7) and one on a headband with the sensor centered on the forehead. These sensors were used to track arm and head movements. Prior to starting the reaching test, the position of the left and right tragus, the medial/lateral and anterior/posterior points of the external support (pelvic or thoracic) and sternal notch were recorded. This allowed estimation of the location of the head center of mass using the center of the distance between the midpoint of the two tragus markers and the head sensor. The center of the trunk region being evaluated was estimated as the midpoint between the sternal notch and C7, and the center of the external support was calculated as the midpoint of the two vectors created by the anterior/posterior and medial/lateral markers of the external support. Position data of all four sensors were referenced to the center of the external trunk support.

#### Electromyography

To determine the mechanisms used by infants to control movement, EMG was recorded via bipolar self-adhesive surface electrodes with poles placed 2–3 cm apart. EMG signals were preamplified (gain × 20), band-pass filtered (10–375 Hz), and then further amplified, sampled at a rate of 1000 Hz per channel, and time-synched with position data. Two dorsal muscle groups and three arm muscle groups were recorded bilaterally (paraspinal muscles at the thoracic spine (T7-8) and lumbar spine (L3-4), at the belly of anterior deltoid, triceps and biceps muscles) in addition to the heart beat (over the 7th intercostal space, below pectoralis major, and over the sternal angle), used during analysis to subtract any heart beat artifacts from the EMGs.

### Data reduction and analysis

#### Video analysis

The video recordings served three purposes. First, the video was used to differentiate between non-directed arm movements and visually guided intentional reaching movements toward the toy. Second, the video was used for classification of the behavior of the movements of the arm during toy presentation. Third, initiation and end of reach were visually analyzed using computerized video-coding software (www.datavyu.org) for further evaluation of the kinematic and EMG parameters. Movements were classified as either (1) pre-reaching movements, also called “spontaneous arm movements,” i.e., oscillating movements of the extended arms or forward directed arm movements (Van der Fits et al., 1999a), (2) unsuccessful reaches: reaching movements not ending in toy contact, associated with a loss of stability and/or requiring support while reaching, and (3) successful reaches: reaching movements ending in toy contact or grasp (De Graaf-Peters et al., 2007). The following types of reaches were not included: (1) the infant initiated a reaching movement toward the toy and lost interest during the trajectory by stopping and looking away; (2) the infant hit the toy; (3) the infant reached with full trunk support, i.e., the infant leaned back against the infant seat prior to reaching; (4) the infant used compensatory strategies like reaching with the head or dragging the toy with the forearm.

All reaches were coded as unimanual or bimanual. We defined bimanual reaches as those in which we visually saw the infant touch the toy with both hands and which also had an onset time difference between both arms of less than 1000 ms. If infants began unimanually and then switched to the other arm before reaching the toy, for the kinematic data analysis, only one arm, considered as the dominant arm, was selected. This selection was the same for bimanual reaches. Arm dominance was determined based on the hand that manipulated the object once it was held.

It is not easy to distinctly determine the start of a goal-directed reaching movement in infants, because one cannot instruct them to start from a defined position or at a given time. Thus, the computerized video-coding program allowed us to determine the onset and offset of all reaches. A light emitting diode (LED), placed on the corner of the visual field, was used to synchronize video and kinematic data during each reaching trial. With this, we were able to select reaches within the trial test time. We defined the onset of a reach as the moment when the infant initiated a movement of the upper extremity toward the toy accompanied by a visual fixation of the target. The offset of the reach was determined when the infant intentionally touched the toy.

To evaluate inter-rater reliability, a second coder scored approximately 25% of the video data. Coders agreed 85.9% of the time on the occurrence of a reach, its type (pre-reach, unimanual or bimanual), κ = 0.87, and whether it was successful or unsuccessful, κ = 0.67. Intra-class correlation coefficient between primary and secondary coders for reach onset and offset times was above 0.90.

After video-coding all reaches, reaching onsets was verified and adjusted, if necessary, by using an interactive cursor display, by simultaneously plotting the XYZ resultant of velocity and position data of the corresponding wrist sensor with the time frame selected with the video. A minimum velocity profile immediately preceding the initiation of the reach, identified from the video-coding software, was then verified. All dependent variables were then calculated from the selected time duration of each reach sequence. Kinematic and EMG data were digitized for off-line analysis with custom MATLAB programs.

#### Kinematic analysis

Kinematic data were filtered with a zero-lag fourth-order low-pass Butterworth filter with a cut-off frequency of 6 Hz to smooth the data and avoid possible jerky movements registered during the reaching sequence. We examined the following variables for each reach: angular trunk displacement and variability of trunk angle during a reach, straightness score, number of movement units, normalized jerk score and time when peak velocity occurred.

The time when peak velocity occurred was calculated as a percentage of time between the onset and end of the reach. A movement unit was defined according to Grönqvist et al. (2011) as the portion of the arm movement between two velocity minima with a velocity peak that should be greater than 2.3 cm/s. If the difference between the highest minima of one movement unit and the peak velocity of another movement unit was less than 8 cm/s, they were considered as one movement unit. Straightness was determined by measuring the trajectory of a straight line from the beginning of the trial to the moment when the infant touched the toy, which is the shortest distance to the target, considered as the baseline path length with a value of one. The amount that the arm movements deviated from this trajectory was then determined as the proportional increase in trajectory compared to this baseline path. Using this method, values greater than one meant a more devious arm movement (Von Hofsten, 1991). The smoothness of the reach was quantified by calculating a time and distance normalized jerk score measured in cm/ms^3^. Time and amplitude were used to normalize the jerk score to eliminate dramatic increases with movement time. The following formula was applied to calculate normalized jerk score,

$$normalized\;jerk\;score=\sqrt{\frac{1}{2}\cdot \int {\left(r^{\prime \prime \prime }\right)}^{2}\text{dt}\cdot (t^{5}/l^{2})}$$

where *r*‴ is the third time derivative of position data, *t* is movement time, and *l* is movement amplitude (Chang et al., 2005).

In terms of postural control, the angular displacement of the trunk was calculated as the angular summation during a reach in the anterior-posterior and medio-lateral planes. The trunk angle was calculated using a vector between the trunk center and the center of external support with respect to the vertical axis. With this, we were able to calculate the standard deviation of the trunk angle during a reach in the anterior-posterior plane. An increase in angular displacement and variability indicates that posture is in disequilibrium.

#### EMG analysis

A frequency domain and Welch's power analyses on randomly selected sessions of the raw EMG signal were used to identify the most appropriate range of EMG signal frequency across the different muscles. Once we identified the most common frequency range, a modified version of the protocol used by Spencer and Thelen (2000) was applied: band-pass filter with cut-off frequencies at 20 and 160 Hz, demean, full-wave rectification and BoxCar averaging with a windows size of 7 data points in order to remove high-frequency components. In addition to this filtering process, a customized algorithm was applied for identifying and subtracting the cardiac QRS-complex signal from each channel of raw EMG before rectification.

Because this study was a within-subject design, the approach used for normalization and identification of EMG bursts was done relative to baseline EMG. This accounts for changes in baseline EMG magnitude and noise within-trials and across conditions for individual participants (William and Adam, 2012). For this purpose, EMG integrals of 10 ms bins were calculated across each muscle signal. A continuous 3 s time window of EMG-baseline signal for each muscle across the entire session was identified and the average integrated EMG of a bin was obtained during this baseline time window (⨛ *EMG*_*Baseline*_). Each EMG integral (⨛ *EMG*_*Integral*_) of a bin was then normalized relative to EMG-baseline bin, $\int EMG_{Norm.Integral}=\frac{\int EMG_{Integral}-\int EMG_{Baseline}}{\int EMG_{Baseline}}$ where ⨛ *EMG*_*Norm.Integral*_ greater than 1 would indicate an increase in EMG activity and less than 1 would indicate inhibition of activity. Thus, for determining significant bursts onsets and offsets, we applied an automatic onset and offset selection: 8 consecutive bins had to have a normalized value of 1.5 or greater (for determining onsets) or smaller (for determining offsets), prior to or during a reach. An interval of 80 ms was used because this time has been shown to be the minimal delay in postural muscle reactions (Horak et al., 1997; Shumway-Cook and Woollacott, 2012).

EMG analysis was structured in two main temporal windows: anticipatory stage, the 500 ms prior to the reaching onset; and compensatory stage which was variable depending on the movement time of the reach (Bigongiari et al., 2011). In comparison to previous studies, we decided to use a larger window size for the pre-defined anticipatory stage because infants, especially during early development, could activate postural muscles well in advance of the reach onset. Frequency of muscle activation during the compensatory stage was calculated as the number of times the EMG signal was active after the reach onset (%EMG_ACTIVATION_ in the compensatory stage). Frequency of muscle activation during the anticipatory stage was calculated as the percentage of times the EMG signal initiated its activation within the 500 ms preceding the reach onset and when its offset occurred at or after the reach onset (%EMG_ACTIVATION_ in the anticipatory stage). For postural muscles, frequency of activation during the compensatory and anticipatory stages was calculated as the percentage of time in which either the thoracic or lumbar muscle was activated. Lastly, we calculated the co-activation rates of the agonist and antagonist muscles of the arm. This was determined as the percentage of trials in which biceps and triceps muscles were simultaneously active with an onset difference of less than 40 ms (Van der Heide et al., 2003).

#### Statistical analysis

Mixed models, in comparison to traditional analysis that do averaging, provide much more flexibility by taking the full data set into account and allowing subjects to have missing time points. Therefore, SPSS 22.0 for Windows (SPSS Inc., Chicago, IL, USA) was used to perform a Generalized Linear Mixed Model (GLMM) analysis of the interrelation between reach outcomes across developmental time and levels of external trunk support. GLMM is an extension of the LMM which allows fitting binary outcomes in addition to continuous outcomes into the model. As fixed effects, we entered developmental time of sitting ability, level of external support (thoracic and pelvic) and also their interaction into the model. As random effects, we had intercepts for infants and for sessions within infants, accounting for by-infant variability and by-session-within-infant variability in overall reach outcomes. Visual inspection of residual plots did not reveal any obvious deviations from homoscedasticity and normality. *Post-Hoc* comparisons using GLMM provided the ability to obtain *post-hoc* pairwise comparisons of the estimated marginal means for different levels of the fixed factors, such as level of external support across developmental time. *P*-values were obtained from *post-hoc* analysis after applying Bonferroni's sequential adjustment procedure that accounted for the multiple comparisons of the model.

## Results

A total of 1730 reaches met the selection criteria. Out of this number, 1587 reaches were successful and were pooled for further kinematic and EMG analysis. Reaching onset occurred between 3 and 4 months of age (*M* = 3.26) when infants were placed in the supine position and were able to successfully contact a graspable toy placed at midline. Sitting onset occurred between 4 and 8 months of age; mean age of sitting onset as defined by item 8 on the AIMS was 5.95 months (Table 1).

### Validity of the SATCo

**Figure 2:**
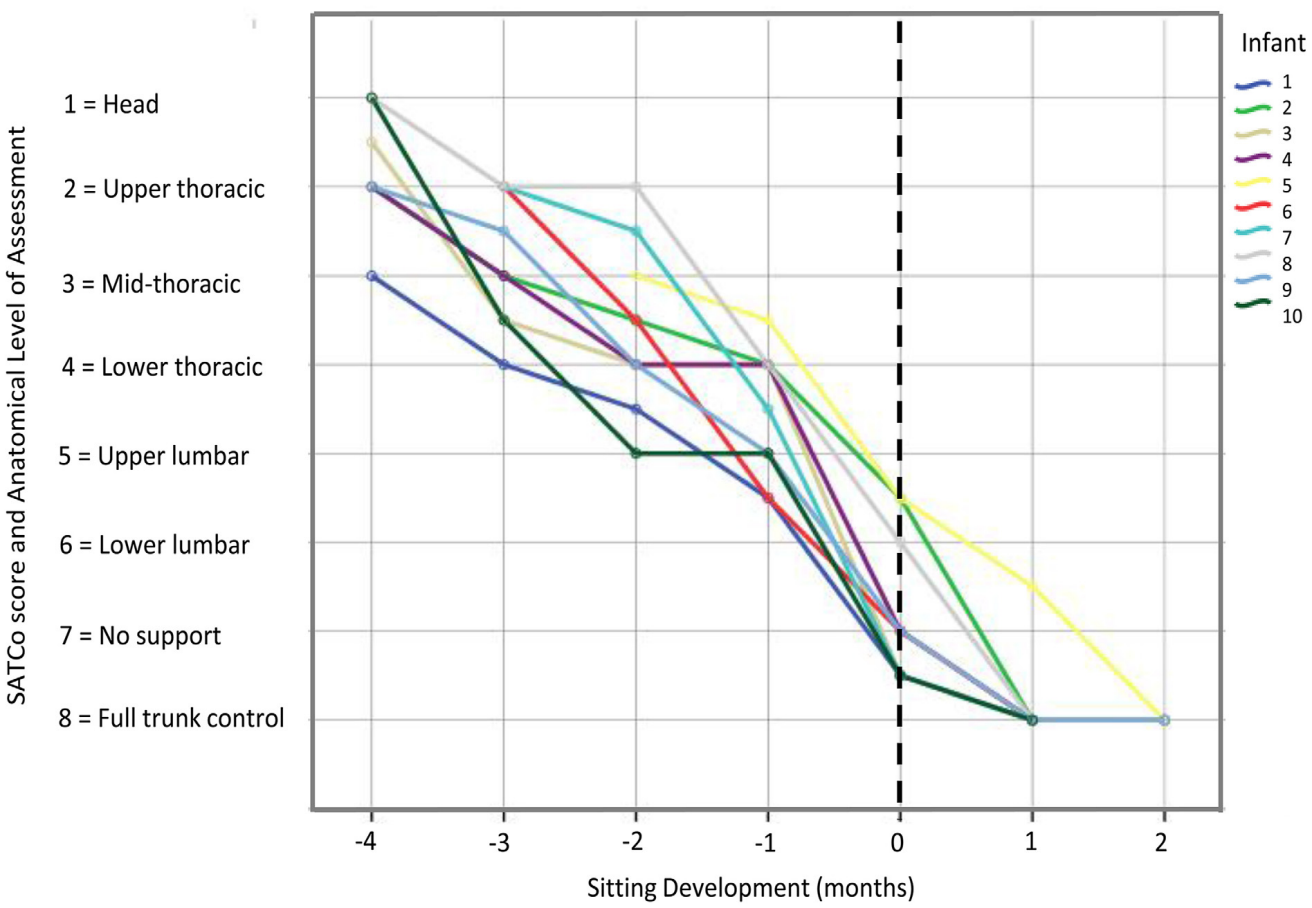
**Graph showing SATCo scores (1–8) across sitting developmental time for each infant**. Vertical dashed line represents time of sitting onset. The developmental time period prior to sitting onset, corresponds to SATCo scores 1 through 5 which was when infants were learning to control progressively the upper and lower trunk regions. Once they acquired the ability to sit independently, it corresponded to SATCo scores 6, 7, and 8, indicating that they had control of almost all trunk segments.

Pearson's product moment correlation coefficient showed high correlation of SATCo scores with: developmental time (*r* = 0.91), AIMS test (*r* = 0.86), Bayley Scales of Infant and Toddler Development test (*r* = 0.83) and age (*r* = 0.90). According to the SATCo test, all infants achieved head control at 3 months prior to sitting onset and eight out of the ten infants achieved complete trunk control at the time of sitting onset (Figure 2).

### Differences in reaching success and type of reach

Four months prior to independent sitting, we were able to examine 7 out of the 10 infants. All attempted to reach toward the toy with thoracic support. The number of attempts was small (*M* = 5 reaches per infant) and the majority were unsuccessful or were classified as pre-reaches (*M* = 3 unsuccessful reaches out of 7 trials). With pelvic support, only 3 out of the 7 infants attempted to reach toward the toy. Most infants could not balance with this level of support and were continuously falling backwards. One infant was unsuccessful during all attempts and the other two were unsuccessful 50% of the time. Thus, for further analysis, infant reaches corresponding to 4 months prior to sitting were not included, due to the limited number of reaching attempts that infants were able to make with the external support at pelvic level.

Then, 3 months prior to sitting, 9 out of the 10 infants attempted to reach with thoracic support, and 8 out of the 10 infants attempted to reach with pelvic support. Infants were still less successful in reaching the toy with pelvic (47% of the time) in comparison to thoracic support (67% of the times), *t*_(152)_ = 2.04, *p* ≤ 0.05, *d* = 0.17. Two months prior to sitting, infants were successful in reaching during approximately all attempts (96% with thoracic support and 86% with mid-rib support) and it was not until 1 month prior to sitting when they were completely successful (100% for both levels of support; see Table 2).

**Table 2:** **Summary of trial data across development**.

	**−4 Months**	**−3 Months**	**−2 Months**	**−1 Month**	**Sitting onset**	**1 Month**	**2 Months**
Number of infants examined	7	9	10	10	10	9	7
Number of sessions	13	14	18	20	20	17	11
Success rate/infant:	40%	67%	96%	100%	100%	100%	100%
thoracic support	(*N* = 7)	(*N* = 9)	(*N* = 10)	(*N* = 10)	(*N* = 10)	(*N* = 9)	(*N* = 7)
Success rate/infant:	18%	47%	86%	100%	100%	100%	100%
pelvic support	(*N* = 3)	(*N* = 8)	(*N* = 10)	(*N* = 10)	(*N* = 10)	(*N* = 9)	(*N* = 7)

*Success rate is based on the average number of times infants were able to successfully touch the toy*.

The type of reach (bimanual vs. unimanual) was variable across developmental time and not related to the level of support provided.

In summary, through the use of video-coding analysis, we were able to clearly distinguish the time when goal-directed reaches started to appear. The number of successful attempts increased with sitting age, but this increase in reaching performance was earlier for thoracic support compared to pelvic support.

### Differences in postural and reaching kinematics across development

**Figure 3:**
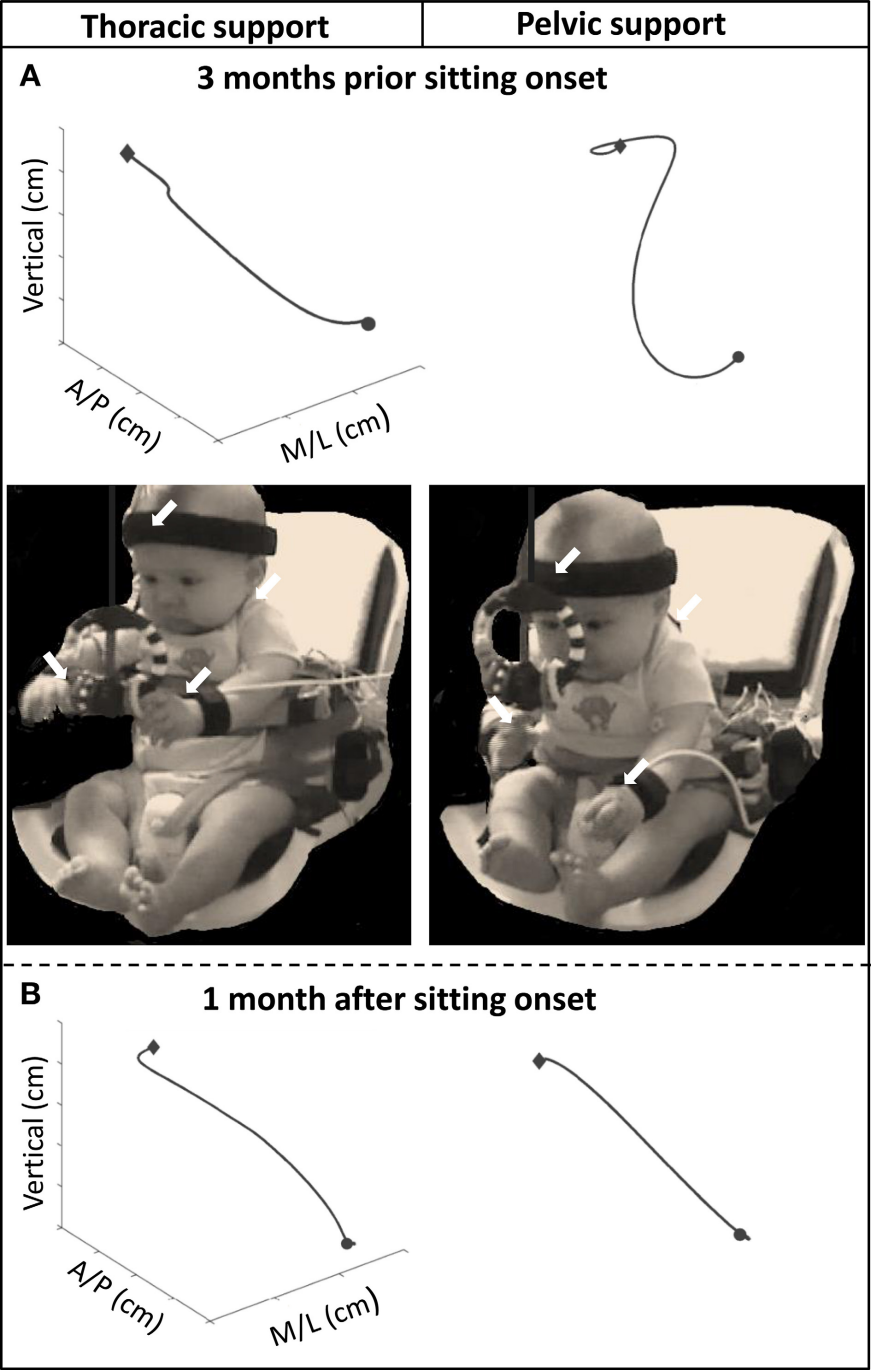
**Graphs above showing examples of the 3D trajectory of a single reach from onset (circular shape) to offset (diamond shape), of one infant with thoracic and pelvic support during (A) 3 months prior to sitting onset and (B) 1 month after sitting onset**. Photographic images show infant reaching toward the toy with thoracic and pelvic support at 3 months prior to sitting onset. Arrows indicate location of kinematic sensors.

Major differences in reach outcomes between levels of external support were observed during the months prior to sitting onset. The graphs from Figure 3 are examples of a reach at the thoracic and pelvic level of support of an infant 3 months before and 1 month after sitting onset. A photographic image is shown of the infant reaching with each level of support at 3 months before sitting onset. The 3-dimensional visual representation of the arm trajectory shows how the infant displayed a more circuitous reach and was more unstable with pelvic support compared to thoracic support prior to the development of independent sitting ability, and this difference was not observed once this milestone was acquired.

**Figure 4:**
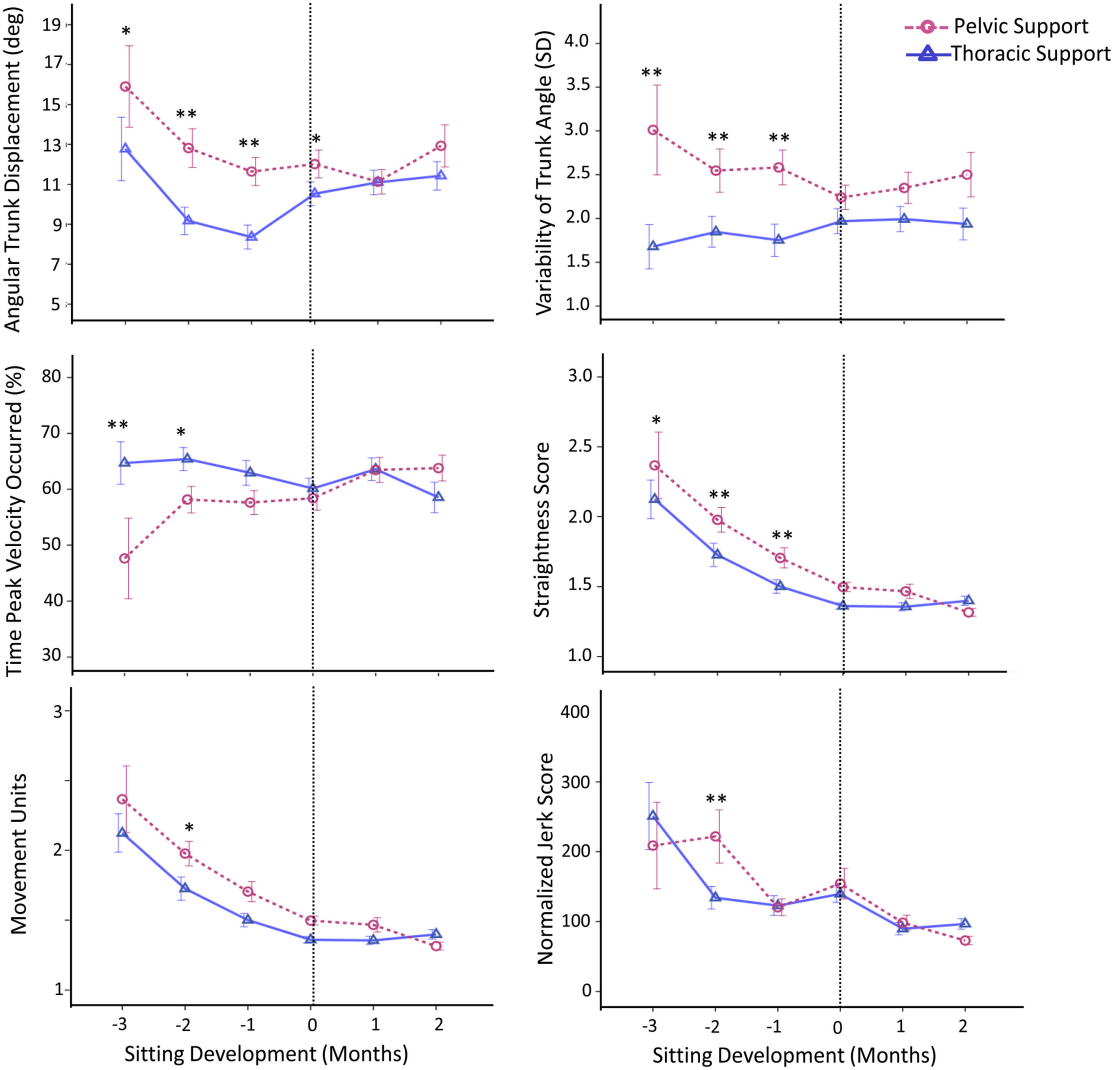
**Estimated means of group data across sitting developmental time**. Y-axes display kinematic variables, X-axes display developmental time in months for thoracic (solid line with triangles) vs. pelvic (dashed line with circles) support. Vertical dotted line represents time of sitting onset. Error bars, ± 1 SE. ^*^p ≤ 0.05, ^**^p < 0.01.

These observations were further corroborated with the kinematic variables (Figure 4). With pelvic support, compared to thoracic support, infants showed an increase in angular trunk displacement at 3 months, *t*_(94)_ = 1.96, *p* ≤ 0.05, *d* = 0.20, 2 months, *t*_(256)_ = 3.78, *p* < 0.01, *d* = 0.24, and 1 month, *t*_(310)_ = 3.41, *p* < 0.01, *d* = 0.19, prior to sitting and at the time of sitting onset, *t*_(344)_ = 2.02, *p* < 0.05, *d* = 0.11. Variability of trunk angle was greater for pelvic support at 3 months, *t*_(94)_ = 3.00, *p* ≤ 0.01, *d* = 0.33, 2 months, *t*_(256)_ = 3.00, *p* < 0.01, *d* = 0.19, and 1 month, *t*_(310)_ = 3.50, *p* < 0.01, *d* = 0.20, prior to sitting.

Reaching kinematics also showed differences between levels of support, being worse with pelvic support. With pelvic support infants showed an increase in: straightness score at 3 months, *t*_(94)_ = 1.92, *p* ≤ 0.05, *d* = 0.20, 2 months, *t*_(256)_ = 3.79, *p* < 0.01, *d* = 0.24, and 1 month, *t*_(310)_ = 2.83, *p* < 0.01, *d* = 0.16, prior to sitting; in movement units at 2 months prior to sitting, *t*_(256)_ = 2.32, *p* < 0.05, *d* = 0.15; and in normalized jerk score at 2 months prior to sitting, *t*_(256)_ = 2.76, *p* < 0.01, *d* = 0.18. Time at which peak velocity occurred was shorter for pelvic support compared to thoracic support at 3 months, *t*_(94)_ = −3.00, *p* < 0.01, *d* = 0.31, and 2 months, *t*_(256)_ = −2.12, *p* < 0.05, *d* = 0.13, prior to sitting.

These kinematic results describe the quality of the motor task and show that with pelvic support compared to thoracic support, maintaining stability of the trunk (measured by angular trunk displacement and variability of trunk angle) was more challenging for infants. However this was only during the period when infants had not yet acquired the ability to independently sit. During the same time period their reaching behavior was worse with pelvic support, as indicated by their straightness score, movement units, normalized jerk score and time to peak velocity.

### Differences in postural and arm EMG

#### Frequency of postural muscle activation

**Figure 5:**
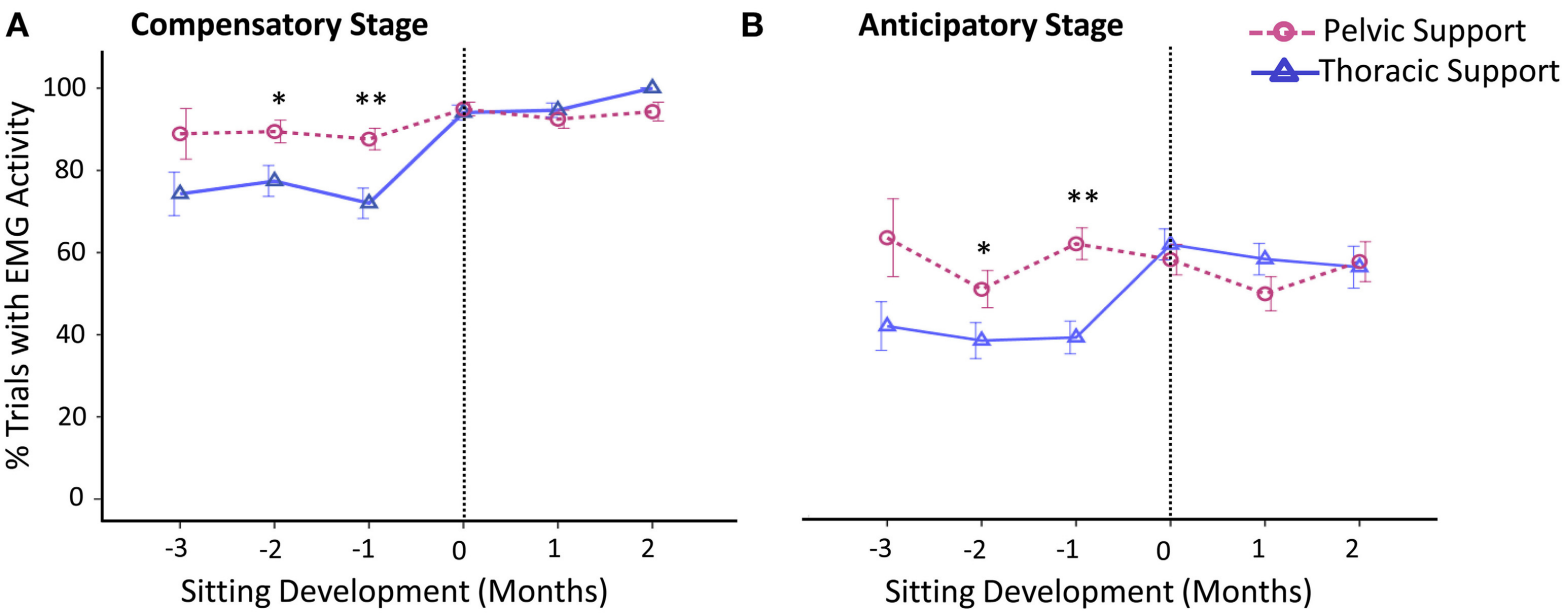
**Estimated means of group data across sitting developmental time**. Y-axis displays percentage of trials with EMG activity during the **(A)** compensatory postural adjustment stage and **(B)** anticipatory postural adjustment stage. X-axis displays developmental time in months for thoracic (solid line with triangles) vs. pelvic (dashed line with circles) support. Vertical dotted line represents time of sitting onset. Error bars, ± 1 SE. ^*^p < 0.05, ^**^p < 0.01.

Differences between levels of external support in frequency of activation of postural muscles were mainly observed during months prior to sitting onset. In general, postural muscles were more frequently activated when infants were supported at pelvic vs. thoracic level and this was not observed once infants acquired independent sitting ability (Figure 5).

In comparison to thoracic support, with the support at pelvic level, infants showed an increased frequency of activation of postural muscles during the compensatory stage at 2 months, *t*_(246)_ = 2.03, *p* < 0.05, *d* = 0.13, and 1 month, *t*_(310)_ = 2.69, *p* < 0.01, *d* = 0.16, prior to sitting. When sitting onset occurred, compensatory adjustments of postural muscles substantially increased with both levels of support.

Similar to the results obtained for frequency of compensatory adjustments, we found that anticipatory postural adjustments were also more often present with pelvic support compared to thoracic support at 2 months, *t*_(246)_ = 2.01, *p* < 0.05, *d* = 0.13, and 1 month, *t*_(310)_ = 4.19, *p* < 0.01, *d* = 0.24, prior to sitting. By the time sitting was achieved, the percentage of anticipatory adjustments had reached similar values for both levels of support. The average onset time of anticipatory adjustments was approximately −285 ms across sitting development, irrespective of support and developmental time.

#### Arm muscle activity

Frequency of activation for the arm muscles was characterized as being highly variable between levels of support and across developmental time; however, in general, results showed that all arm muscles were consistently active during a reach, biceps activity being the most predominant.

**Figure 6:**
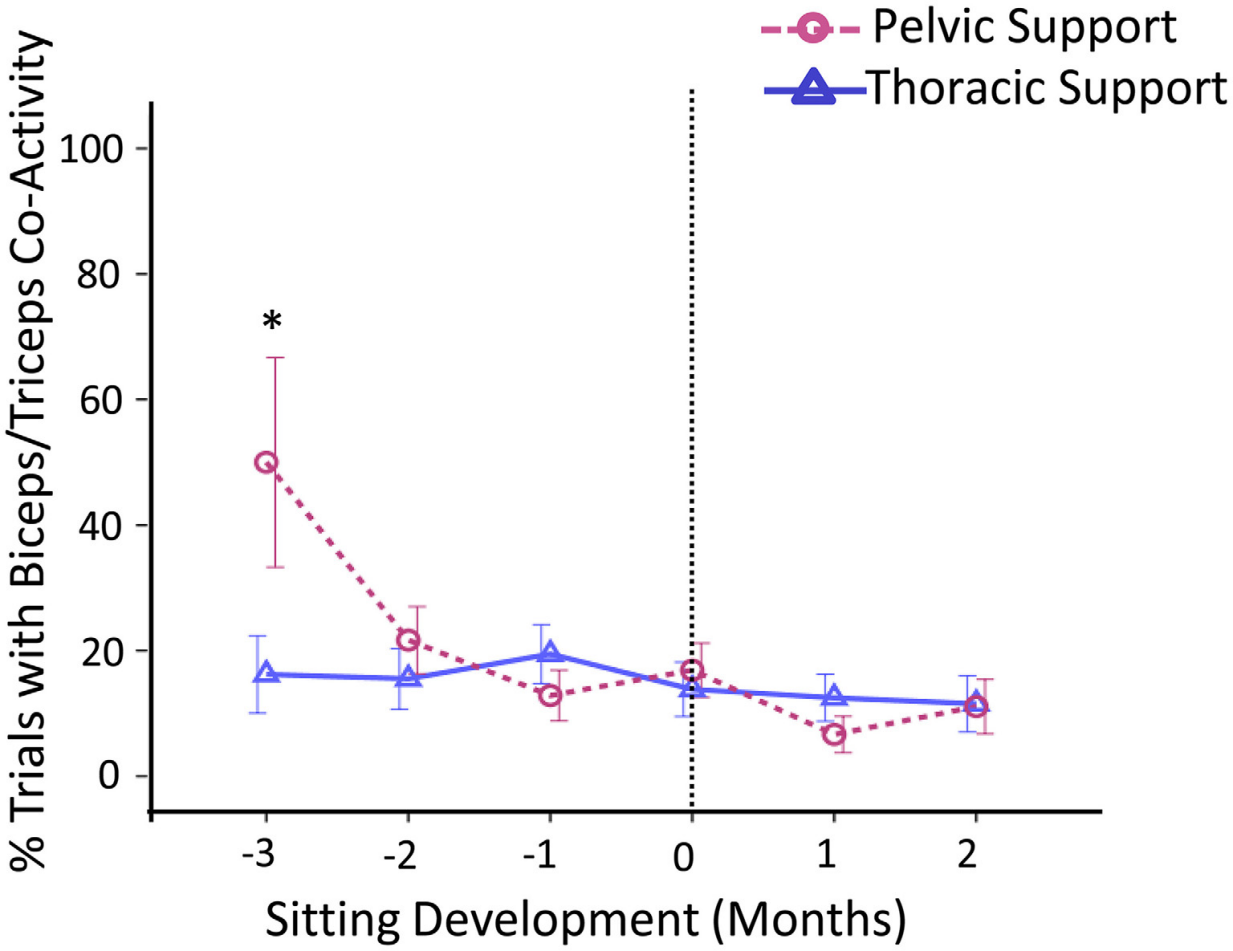
**Estimated means of group data across sitting developmental time**. Y-axis displays percentage of trials with co-activity of biceps and triceps muscles. X-axis displays developmental time in months for thoracic (solid line with triangles) vs. pelvic (dashed line with circles) support. Vertical dotted line represents time of sitting onset. Error bars, ± 1 SE. ^*^p < 0.05.

Co-activation rates for biceps-triceps activity were substantially higher for pelvic support compared to thoracic support 3 months prior to sitting, *t*_(46)_ = 2.00, *p* < 0.05, *d* = 0.29 (Figure 6).

Overall, through the use of EMG we were able to document the mechanisms of change in seated reaching across development. As with kinematics, results indicate that pelvic support was more challenging than thoracic support during the period when infants have not yet acquired independent sitting. This was determined by the increase in activation frequency of postural muscles, during both anticipatory and compensatory stages, and increase in co-activation rates of the arm muscles at 3 months prior to sitting onset.

## Discussion

This study was motivated by the hypothesis that the development of sitting postural control and reaching behavior are highly interdependent functions. Full attempts were made to tease out the causal effects of postural control on reaching. First, having applied measures across a broad range of ages in a longitudinal design, we were able to explore a critical window of postural development prior to independent sitting. Second, with the use of experimental manipulations, we had the means to model the type of postural control that infants progressively generated for themselves. We provided external support to the thoracic and pelvic regions of the trunk to compare the effects of increased vs. decreased postural support on reaching.

With support at the thoracic level, we confirm and expand previous results by showing that reaching movements during pre-sitting stages were smoother, more coordinated and more mature than when support was limited to the pelvic level (Rochat and Goubet, 1995; Hopkins and Rönnqvist, 2002; De Graaf-Peters et al., 2007; Rachwani et al., 2013). Increased postural support had a direct impact on reaching performance and neuromuscular responses of the trunk. As infants developed trunk control, they no longer required higher support to produce coordinated reaching. The data indicate that postural control is a primary factor contributing to reaching proficiency, regardless of whether posture improves naturally across age or with the help of an experimental set-up (Adolph and Berger, 2005). This information creates the basis for future studies that can be applied in assessment and rehabilitative protocols in children with postural dysfunctions.

### Reaching success

Infants at early stages of sitting development (4 months prior to sitting onset), showed minimal ability to remain stable in the sitting position when provided with thoracic support. This ability was completely absent in most infants with pelvic support, in which only 3 infants were able to maintain stability part of the time. Similar results were seen with respect to the number of reaching attempts that the infants made with the two levels of support. Thus, even though both motor milestones, upright sitting and reaching, were still immature during this developmental time period, better support of the trunk was associated with the ability to maintain stability and to perform more reaches, as has been observed in previous studies (Von Hofsten, 1982; Amiel-Tison and Grenier, 1983). This suggests that postural control significantly regulates the interaction of the infant with the surrounding environment during development, facilitating new actions, like reaching, which can promote the emergence of cognitive skills and social behaviors (Gibson, 1988).

According to the AIMS and SATCo scores, infants began to master head control at 3 months prior to sitting onset. With this mastery, infants increased their ability to touch/grasp the toy, highlighting the importance of head control for successful reaching (Thelen and Spencer, 1998). In order to lift the arm and successfully touch the toy infants must fixate the visual target, which requires both strength and control of the head in space. We hypothesized that reaching abilities would be reduced when postural stability was reduced (i.e., with pelvic support) and, as predicted, infants were more successful in reaching with thoracic compared to pelvic support. Then, at 2 and 1 month prior to sitting onset, the success rate was similar between conditions, despite the challenging postural demands derived from trunk support at the pelvic level. Harbourne et al. (2013) showed a similar effect in that non-independent sitters persistently and successfully reached in spite of subsequent falls, disorganized muscle onsets and erratic trunk movements.

### Postural and reaching kinematics

The effect of external support on the control of posture while reaching was evident in that infants showed reduced stability with pelvic support when they had not yet mastered the ability to sit independently. Postural sway was quantified as total displacement of the trunk and angular variability while reaching. With thoracic support, posture was more controlled and subsequent reaching performance was better during pre-sitting stages. Reaching movements performed under the high support (thoracic) condition were straighter, (smaller straightness score), smoother (fewer movement units), more efficient (less jerk score) and used a more refined program (greater percentage of reach time when peak velocity occurs) than those performed under the low support (pelvic) condition.

The point in time at which differences between support levels disappeared depended on the kinematic variable measured. For instance, differences between support levels in smoothness, efficiency and programming of a reach were seen only at 2 months prior to sitting and then disappeared. This indicates that other factors may contribute to further kinematic improvement. The straightness of a reach on the contrary, was persistently affected by support level during all pre-sitting ages with infants generating more circuitous reaches with pelvic support. Being able to independently sit marked the hallmark for performing straight, linear reaches regardless of the support level. Taken together, these results suggest that the ability to produce efficient and accurate reaching in sitting is due to maturation of trunk control.

### Postural and reaching EMG patterns

On numerous occasions, researchers showed that postural muscle activity accompanying reaching movements increases with age (De Graaf-Peters et al., 2007; Van Balen et al., 2012; Harbourne et al., 2013). Results from the current study show that postural muscle activity can be present even in early stages of sitting development, but it is dependent on the constraints of the task. Compensatory postural muscle activity was more frequent when infants were provided with pelvic support 2 and 1 month prior to sitting onset. This implies that during sitting development, infants were able to recruit postural muscles while reaching, and to increase recruitment frequency when the postural task was more demanding. Thus, postural muscle recruitment was situation-specific and depended on the degree of instability (Hadders-Algra, 2008). Then, with increased age and maturation of sitting ability, the activation frequency of postural muscles increased and infants showed similar values between levels of trunk support, implying that pelvic support no longer produced instability. Previous research showed that once independent sitting was mastered, postural muscle activity accompanying reaching movements while sitting was consistently present (Van der Heide et al., 2003) and thus became embedded in the task, although it could be further enhanced if the risk of losing balance was high (Hadders-Algra, 2005; Van Balen et al., 2012).

Similarly, though anticipatory adjustments were just emerging in pre-sitting stages, infants displayed a higher percentage of anticipatory postural adjustments when they were provided with pelvic support compared to thoracic support, indicating they were anticipating the disequilibrium the reaching created when they had not yet acquired full trunk control. After the time of sitting onset, anticipatory adjustments were more consistently present (more than 50% of the time) and were independent of the type of support, suggesting this was related to the onset of independent sitting. The study by Van der Fits et al. (1999b) examining anticipatory postural adjustments under conditions of upright sitting concluded that anticipatory postural adjustments were present only inconsistently (20% of trials) at 6 months of age and became more regular at 13–14 months. These differing results might be explained by methodological differences related to the time period that was selected for analysis, because Van der Fits et al. (1999b) evaluated 200 ms prior to prime mover activation whereas in the current study we evaluated 500 ms prior to reach onset. To test our theory, we re-evaluated our data with a 200 ms window and found a 20% frequency of anticipatory postural adjustments across development and support level. The smaller window replicates the findings of Van der Fits et al. (1999b) and eliminates the ability to see the developmental pattern, thus demonstrating the critical importance of window size when making conclusions about developmental trajectories.

In short, during early developmental stages of sitting, anticipatory postural adjustments accompanying reaching movements are present to some degree, especially when the postural task is more demanding. However, they are characterized by immature temporal features. Anticipatory postural adjustments start to play a major role in the postural mechanisms for seated reaching once independent sitting has been established. At this point their activation is not dependent on the level of postural stability but they are consistently activated well in advance (approximately 285 ms prior to reach onset).

The activation of both agonist and antagonist muscles at a joint often occurs when the individual has lower skill levels because co-activation stiffens the entire limb. In this study the co-activation rates of biceps and triceps muscles were significantly enhanced with pelvic support during early stages of sitting control, which was also associated with the onset of reaching (Thelen et al., 1993). This outcome implies the need to maintain arm stability when seated conditions had increased postural requirements. Nevertheless, our findings related to arm muscles indicate that frequency of activation was highly consistent and was not dependent on the level of support. This could be explained by the following reasons: first, infant arm movements were seldom at rest and therefore arm muscles were often active even prior to the start of the reach. Second, because infants were in upright sitting conditions, they showed arm movements that were not related to the reach but were used as compensatory strategies to maintain balance. For these reasons, the starting point of the reach was not identical across trials despite the attempts made to avoid this.

To conclude, results reinforce and further expand previous findings showing that improvements in sitting control have direct consequences on the development of reaching. There is a cranio-caudal acquisition of trunk control for independent sitting. The extent of sitting control acquired has an impact on the kinematic quality of reaching movements and accompanying postural muscle patterns, attributed to frequency of activation. However, with additional support, infants experience improvements in their reaching skills and subsequent muscular parameters during the development of upright sitting. Further research should examine differences in compensatory balance strategies and muscle response patterns used to recover from seated perturbations with different levels of trunk support. Moreover, the interrelation of reaching and sitting postural control should also be examined in children with cerebral palsy to determine if they might benefit from external trunk support and consequently implement more efficient therapeutic strategies. This paradigm offers the foundation for future exploration both in typical development and in children with neurological deficits.

### Conflict of interest statement

The authors declare that the research was conducted in the absence of any commercial or financial relationships that could be construed as a potential conflict of interest.
