# Corrigendum: The development of trunk control and its relation to reaching in infancy: a longitudinal study

**DOI:** 10.3389/fnhum.2015.00406

**Published:** 2015-07-21

**Authors:** Jaya Rachwani, Victor Santamaria, Sandra L. Saavedra, Marjorie H. Woollacott

**Affiliations:** ^1^Department of Human Physiology and Institute of Neuroscience, University of OregonEugene, OR, USA; ^2^Department of Rehabilitation Sciences, University of HartfordWest Hartford, CT, USA

**Keywords:** infant development, independent sitting, posture, trunk control, reaching, EMG

Figure 4 of the article by Rachwani et al. (2015) contained a minor error, which we hereby rectify. In the original figure, the graph displaying the number of movement units across sitting development is incorrect (bottom graph on the left column). We therefore re-submit Figure 4 with the correct graph. We sincerely apologize for the inconvenience.

**Figure 4:**
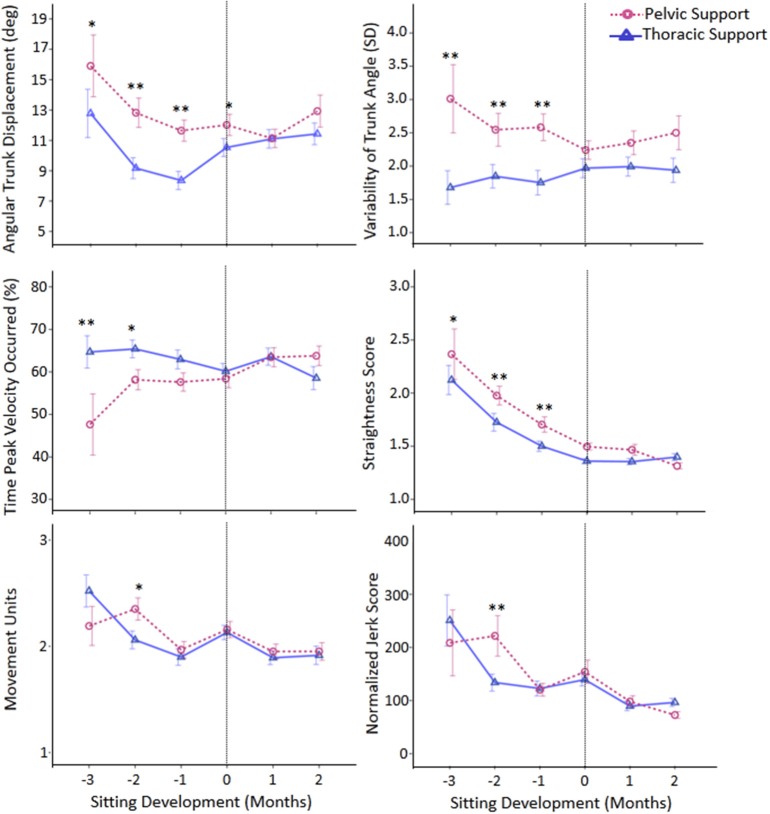
**Estimated means of group data across sitting developmental time**. Y-axes display kinematic variables, X-axes display developmental time in months for thoracic (solid line with triangles) versus pelvic (dashed line with circles) support. Vertical dotted line represents time of sitting onset. Error bars, ±1 SE. ^*^p ≤ 0.05, ^**^p < 0.01.

## Conflict of interest statement

The authors declare that the research was conducted in the absence of any commercial or financial relationships that could be construed as a potential conflict of interest.
